# A case report of immunoglobulin M nephropathy manifesting as crescentic glomerulonephritis and nephrotic syndrome in an adult

**DOI:** 10.1186/s12882-019-1528-2

**Published:** 2019-08-27

**Authors:** Kyoung Sook Park, Ea Wha Kang, Jeong Hae Kie

**Affiliations:** 1 Department of Internal Medicine, Division of Nephrology, NHIS Ilsan Hospital, Goyang, Gyeonggi-do, 10444 Republic of Korea; 2 Department of Pathology, NHIS Ilsan Hospital, Goyang, Gyeonggi-do, 10444 Republic of Korea

**Keywords:** Immunoglobulin M nephropathy, Crescentic glomerulonephritis, Nephrotic syndrome

## Abstract

**Background:**

The nature of immunoglobulin M (IgM) nephropathy has been controversial for a long time, but it is now considered an independent disease like immunoglobulin A nephropathy. IgM nephropathy has been known to have various clinical manifestations ranging from asymptomatic hematuria and/or proteinuria to nephrotic syndrome. Recently, one case of IgM nephropathy manifesting as crescentic glomerulonephritis (GN) was reported in a child.

**Case presentation:**

We experienced a case of IgM nephropathy that manifested clinically as nephritic and nephrotic syndrome with pathologically confirmed crescentic GN in a 30-year-old woman. We administered a calcineurin inhibitor and corticosteroids to treat the ongoing nephrotic syndrome after remission of crescentic GN. As a result, her proteinuria was significantly reduced and edema improved.

**Conclusions:**

We described a case of IgM nephropathy in an adult patient who initially developed crescentic GN with nephritic and nephrotic syndrome. This case report could contribute to a deeper understanding of IgM nephropathy.

## Background

Based on two seminal reports in the 1970s [1, 2], immunoglobulin M (IgM) nephropathy has been defined as IgM deposition in the mesangium, characterized by diffuse and granular patterns [3, 4]. As IgM nephropathy has been clinically found to share some characteristics with minimal change disease (MCD) and focal segmental glomerulosclerosis (FSGS), it has been controversial whether IgM nephropathy can be classified as an independent disease entity. Recently, some studies showed evidence that IgM nephropathy has distinctive features from those of MCD [5, 6]. While IgM nephropathy is known to have a broad range of clinical manifestations, from asymptomatic hematuria and/or proteinuria to nephrotic syndrome [7–9], only one case manifesting as crescentic glomerulonephritis (crescentic GN), which was found in an 11-year-old Pakistani girl, was reported in terms of pathological characteristics [10]. However, the clinical course and treatment of this case were not specified because of failure in follow-up.

Here, we describe the case of a 30-year-old woman diagnosed with IgM nephropathy that manifested as acute nephritic and nephrotic syndrome with pathologically confirmed crescentic GN.

## Case presentation

**Fig. 1:**
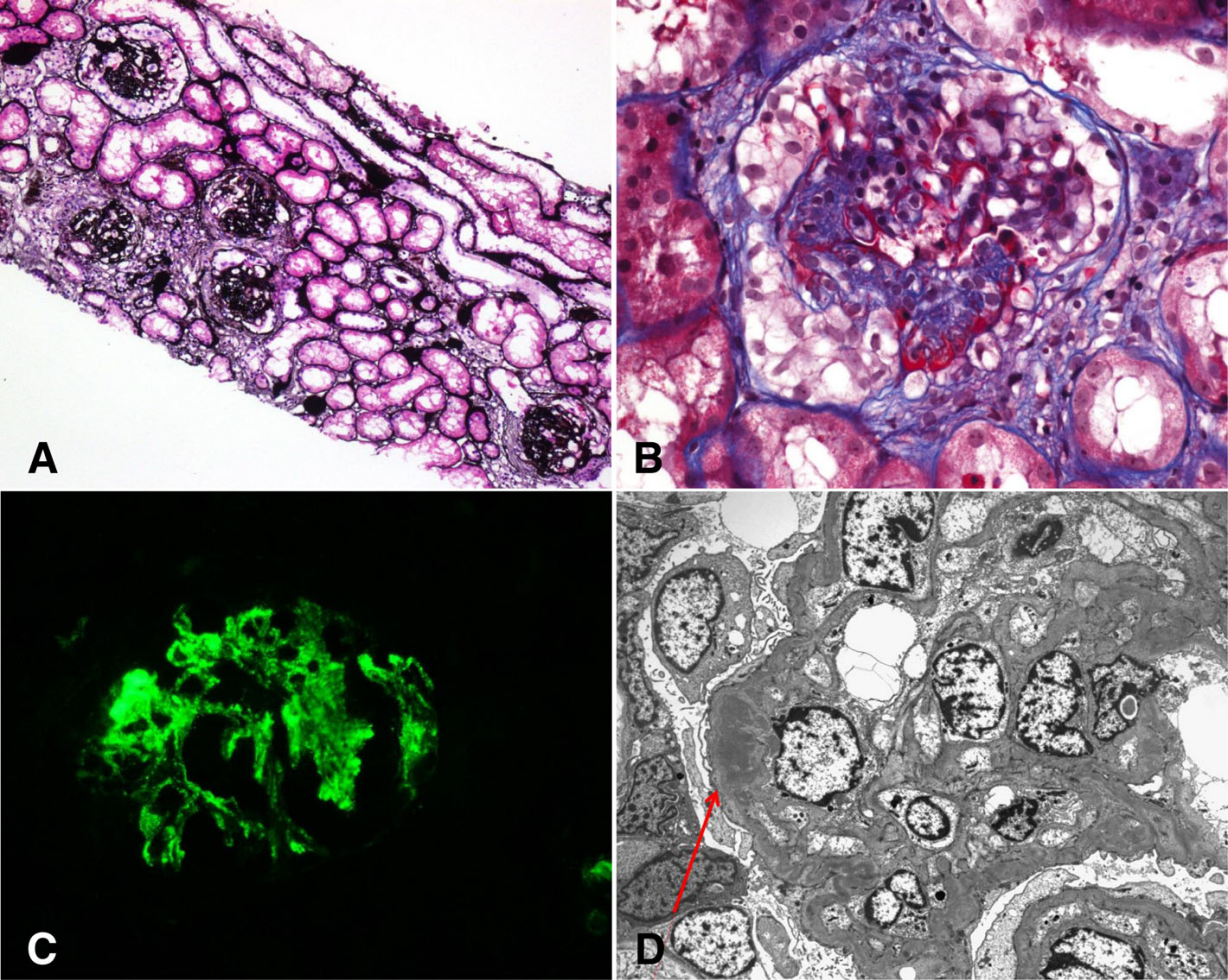
Histological findings of the first biopsy. **a** At lower power view, cellular (left upper & lower) and fibrocellular (right lower) crescents are observed (PAM, × 100) **b** At high power view, a cellular crescent showing fibrinoid necrosis and focal destruction of Bowman’s capsule with periglomerular inflammatory reaction is observed (TRC, × 400). **c** Global immunofluorescent activity for immunoglobulin M can be observed in the mesangium (original magnification × 400). **d** Electron microscopic examination showing electron-dense deposits in the mesangium (red arrow) with mesangial proliferation (uranyl acetate, original magnification × 2500).

A 30-year-old woman visited the nephrology clinic because of proteinuria and hematuria. The patient presented with generalized edema, and her urine had been foamy for 2 months. Her medical history was unremarkable. Her blood pressure was 112/60 mmHg, and pitting edema was observed during her physical examination. Abdominal ultrasonography revealed that both kidneys had a normal size and echogenicity. In our initial laboratory tests, the following values were found: hemoglobin level, 11.6 g/dL; platelet count, 322 × 10^3^/μL; total protein level, 5.1 g/dL; serum albumin level, 2.01 g/dL; serum creatinine level, 1.14 mg/dL (corresponding to an estimated glomerular filtration rate [eGFR] of 64.9 mL/min/1.73 m^2^); and total cholesterol level, 395 mg/dL. Urinalysis revealed proteinuria (2+) and hematuria (2+; red blood cell count, > 20/high-power field). The spot urine protein-to-creatinine ratio (UPCR) was 7.32 g/g, and the spot urine albumin-to-creatinine ratio was 5.24 g/g, with nonselective glomerular proteinuria detected by urine electrophoresis. The results of other serological tests were positive for the antinuclear antibody (1:160), and negative for a speckled pattern and anti-neutrophil cytoplasmic antibody (ANCA); additionally, her serum complements were within their reference ranges. All viral serological markers were negative. Kidney biopsy was performed. Twenty of 30 glomeruli showed cellular or fibrocellular crescents with mesangial proliferation. There was focal mild to moderate acute and chronic inflammatory cell infiltration with mild interstitial fibrosis and tubular atrophy, mainly along the crescentic glomeruli. There was occasional fibrinoid necrosis in the crescentic glomeruli, but there was no vasculitis in the interstitium. Some glomeruli showing mesangial proliferation or segmental sclerotic change without fibrinoid necrosis were observed. The immunofluorescent study revealed diffuse global immunofluorescent activity for IgM (1+), immunoglobulin G (IgG) (trace), and complement 3 (C3) (trace) in the mesangium. The corresponding electron-dense deposit was confirmed by the electron microscopic (EM) examination (Fig. 1). A diagnosis of immune complex-mediated crescentic GN, possibly IgM nephropathy, was made.

As a treatment regimen for crescentic GN, three pulses of intravenous methylprednisolone (750 mg/day) were administered, followed by daily oral prednisone and intravenous cyclophosphamide (500 mg/m^2^ every 3 weeks). Her serum creatinine level continuously increased to 1.66 mg/dL (eGFR, 36.5 ml/min/1.73 m^2^) after admission and then decreased to 1.09 mg/dL (eGFR, 59.3 ml/min/1.73 m^2^) when she was discharged on the eighth day of admission. Three months later, her serum creatinine level was stabilized at 1.11 mg/dL (eGFR, 57.7 ml/min/1.73 m^2^), and microscopic hematuria completely disappeared. However, she steadily complained of generalized edema, and considerable nephrotic-range proteinuria persisted at a spot urine UPCR of 7.73 g/g. Given the tendency of resistance to cyclophosphamide treatments, rituximab was administered intravenously (375 mg/m^2^ per week) for 4 weeks alternatively. After the first dose of rituximab, her UPCR decreased to 0.27 g/g. However, after 4 weeks of rituximab therapy, the UPCR increased again to 5.59 g/g.

**Fig. 2:**
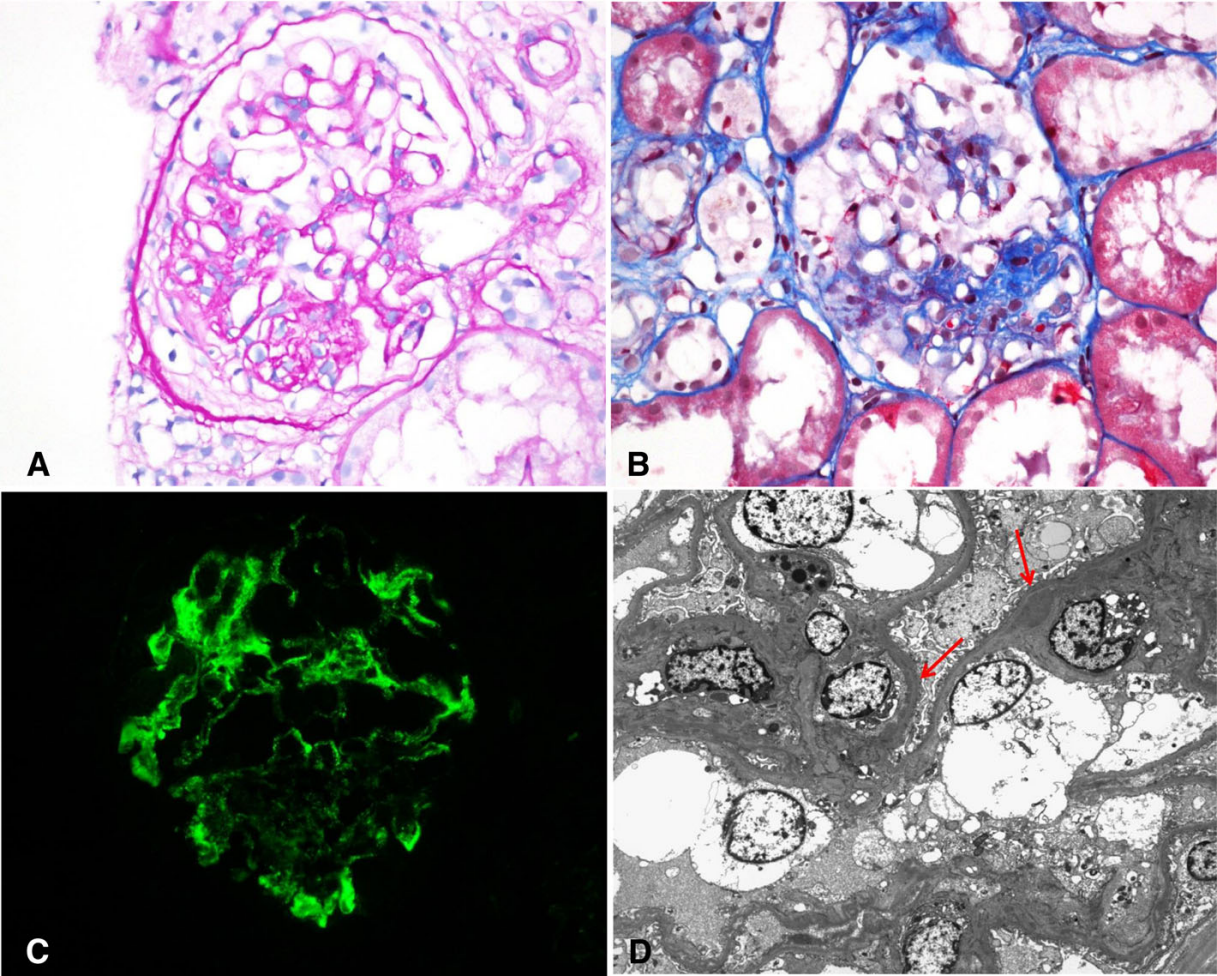
Histological findings of the second biopsy. **a** A glomerulus showing segmental sclerosis with a fibrous crescent (periodic acid methenarnine silver, original magnification × 400). **b** Another glomerulus showing segmental sclerosis without crescents (TRC, original magnification × 400). **c** Global immunofluorescent activity for immunoglobulin M is observed in the mesangium (original magnification × 400). **d** Electron microscopic examination showing electron-dense deposits in the mesangium (red arrows; uranyl acetate, original magnification × 2500)

**Fig. 3:**
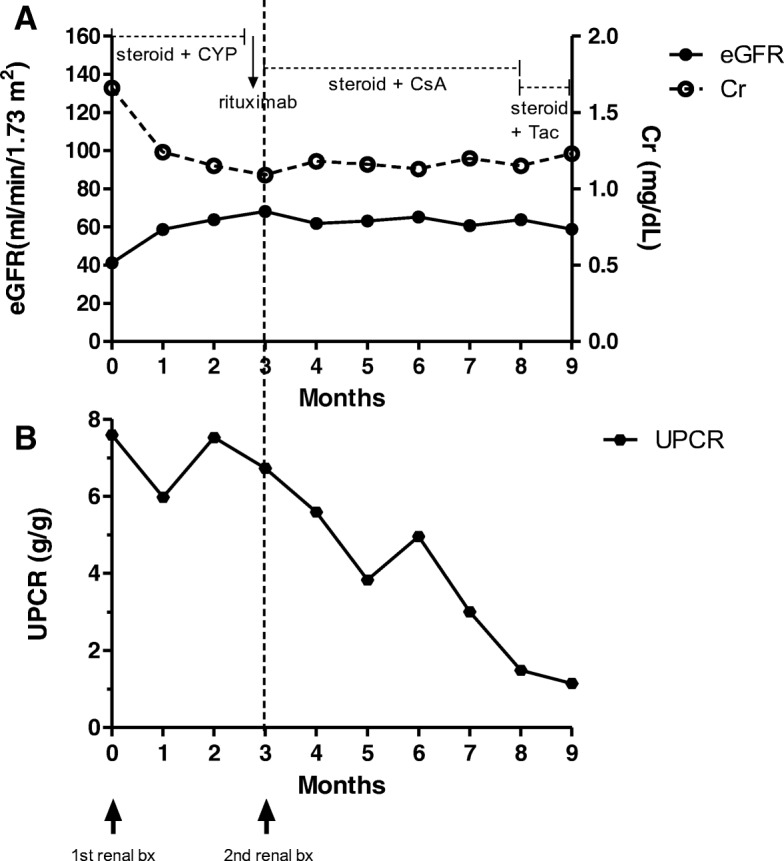
Clinical course of the patient. **a**) Estimated glomerular filtration rate (estimate glomerular filtration rate, left y-axis) according to the Chronic Kidney Disease Epidemiology Collaboration equation. The serum creatinine concentration is also shown (mg/dL, right y-axis). **b**) Urine protein-to-creatinine ratio (left y-axis). On the x-axis, the time after the first renal biopsy within 1 month is shown Abbreviations: CYP, cyclophosphamide; CsA, cyclosporine; Tac, tacrolimus; Cr, creatinine.

A second kidney biopsy was performed to make a precise diagnosis and to evaluate the change in glomeruli after the former treatment. Ten of 13 glomeruli showed segmental sclerosis with occasional mesangial proliferation, and most of them showed fibrous crescents. Mild interstitial fibrosis with tubular atrophy and minimal lymphoplasma cell infiltration were found. Diffuse global immunofluorescent activities for IgM (1+), IgG (trace), and C3 (trace) were observed, with electron-dense deposits in the mesangium, like in the first biopsy (Fig. 2). A diagnosis of IgM nephropathy with a resolving phase of crescentic GN was made. After the second renal biopsy, cyclosporine was administered to treat the nephrotic-range proteinuria, and the UPCR in a spot urine sample decreased from 5.59 g/g to 2.24 g/g. The patient also reported an improvement in edema. Two months after the cyclosporine therapy, the serum creatinine level was 1.37 mg/d (eGFR, 51.6 ml/min/1.73 m^2^) and spot urine UPCR was 1.49 g/g. Owing to the risk of nephrotoxicity, we replaced cyclosporine with tacrolimus. At the recent follow-up, renal function and proteinuria were stabilized with a serum creatinine level of 1.23 mg/dl (eGFR, 58.8 ml/min/1.73 m^2^) and UPCR of 0.88 g/g, without symptoms of edema (Fig. 3).

## Discussion and conclusions

This is the first case report of an adult patient who was diagnosed with IgM nephropathy manifesting as crescentic GN and nephrotic syndrome. The pathological findings of IgM nephropathy have been diversely reported to date, from almost normal histological findings such as MCD to findings such as FSGS. Connor et al. [9] recently studied 57 cases of adult IgM nephropathy to identify its clinical and pathological characteristics based on the following histological criteria. (1) Dominant staining with immunofluorescence or immunoperoxidase for IgM must be present in the glomeruli; the intensity of the IgM staining (graded on a semiquantitative scale) should be more than trace; the distribution of the IgM staining should include the mesangium, with or without capillary loop staining; immunoglobulin A (IgA) and IgG may be present but not in equal or greater intensity than that of IgM; and C3 and C1q may both be present. (2) Definite mesangial deposits must be found in the EM examination. (3) There should be no evidence of systemic disease (e.g., systemic lupus erythematosus, rheumatoid arthritis, diabetes mellitus, and paraproteinemia). In the first biopsy, > 50% of cellular crescents indicated the definitive diagnosis of IgM nephropathy difficult. However, IgM immunofluorescence activity and electron-dense deposition in the mesangium with mesangial proliferation or segmental sclerotic change in non-crescentic glomeruli made us suspicious of the possibility of IgM nephropathy. In the second biopsy, only fibrous crescents were observed owing to remission of the cellular crescents, and IgM nephropathy could be diagnosed because the segmental sclerotic change or mild mesangial cell proliferation was still observed in the non-crescentic glomeruli with diffuse global immunofluorescent activity for IgM (1+) and electron-dense deposition in the mesangium. The significance of mesangial IgM deposition in IgM nephropathy has been controversial [11], and only a few reports have indicated that abnormalities in circulating IgM may play a role, as has been established for IgA nephropathy [12–14]. Previously, many investigators have suggested that IgM nephropathy converts to FSGS over time because most repeated biopsy samples in patients with relapse of nephrotic syndrome or deteriorating renal function showed histological characteristics of FSGS [7, 9, 15]. However, IgM nephropathy is different from FSGS because diffuse mesangial IgM positivity is continuously maintained. In our case, segmental sclerosis with diffuse global immunofluorescent activity for IgM in the mesangium was also observed on repeated biopsy. Another reasonable clue of the pathogenic role of IgM is that IgM nephropathy recurred in a transplant patient [9, 16, 17].

Regarding the clinical manifestation of IgM nephropathy, subnephrotic proteinuria and nephrotic syndrome were commonly observed in the studies involving adults, but no patient presenting with crescentic GN has been reported [7–9]. Although a few studies have reported small subcapsular crescents in cases of IgM nephropathy, to date, only one case of IgM nephropathy manifesting with full-blown crescentic GN has been reported in a child [10]. In a long-term follow-up study of IgM nephropathy in adults, investigators excluded four patients who met the criteria for IgM nephropathy at the time of the first biopsy but subsequently developed an immune complex or ANCA-associated GN [9]. These cases may coincide with our case in clinical and pathological aspects. These cases, including ours, suggest that the spectrum of IgM nephropathy is wider than that known in the literature and closely resembles IgA nephropathy, a well-known immune complex-mediated disease, not only clinically but also morphologically. The presence of diffuse mesangial IgM deposits in our case suggests that IgM nephropathy can also manifest immune complex-mediated crescentic GN such as mesangial IgA deposits in crescentic GN associated with IgA nephropathy.

Our patient was treated initially with pulse methylprednisolone followed by daily oral prednisone and intravenous cyclophosphamide as empirical therapy for idiopathic crescentic GN. After treatment, renal function was stabilized, but nephrotic-range proteinuria continued. On the basis of reports on the therapeutic effect of rituximab in IgM nephropathy [16, 17], we administered rituximab. However, because of the insufficient effect of rituximab, a calcineurin inhibitor was administered, and proteinuria was shown to be partially remediated. The treatment of IgM nephropathy has not been fully elucidated. In most cases, corticosteroid therapy was used first as an immunosuppressive agent for patients presenting with nephrotic syndrome in IgM nephropathy. However, the response rate to corticosteroid therapy ranges from only 20 to 30% [7, 9]. Considering the resistance of IgM nephropathy to corticosteroid treatment, several reports have examined the efficacy of alternative immunosuppressants such as calcineurin inhibitors and rituximab. In prior studies, the treatment-response rate to oral cyclophosphamide was 50% [7], and the treatment-response rates to rituximab and cyclosporine were relatively higher [11, 16–18].

In addition to the previous case report involving a girl in Pakistan, this case report provides evidence of IgM nephropathy manifesting as crescentic GN, particularly in an adult. This case report will help expand physicians’ knowledge on the clinical and pathological spectrum of IgM nephropathy. For a better understanding of IgM nephropathy, more evidence is needed from studies with a larger number of patients and longitudinal research designs.

## Data Availability

Data regarding this study were obtained from clinical charts stored in the physician office records of National Health Insurance Medical Center (NHIMC) and the tissue bank of NHIMC-Biobank and, therefore, cannot be shared. Any reasonable request to access the data must be approved before the data can be released.

## Abbreviations

ANCA: Anti-neutrophil cytoplasmic antibody
C3: Complement 3
eGFR: Estimated glomerular filtration rate
EM: Electron microscopy
FSGS: Focal segmental glomerulosclerosis
GN: Glomerulonephritis
IgG: Immunoglobulin G
IgM: Immunoglobulin M
MCD: Minimal change disease
UPCR: Urine protein-to-creatinine ratio

## References

[1] Cohen AH, Border WA, Glassock RJ (1978). Nehprotic syndrome with glomerular mesangial IgM deposits. Lab Investig.

[2] Bhasin HK, Abuelo JG, Nayak R, Esparza AR (1978). Mesangial proliferative glomerulonephritis. Lab Investig.

[3] Hirszel P, Yamase HT, Carney WR, Galen MA, Graeber CW, Johnson KJ (1984). Mesangial proliferative glomerulonephritis with IgM deposits. Clinicopathologic analysis and evidence for morphologic transitions. Nephron.

[4] Brugnano R, Del Sordo R, Covarelli C, Gnappi E, Pasquali S (2016). IgM nephropathy: is it closer to minimal change disease or to focal segmental glomerulosclerosis?. J Nephrol..

[5] Border WA (1988). Distinguishing minimal-change disease from mesangial disorders. Kidney Int.

[6] Vanikar A (2013). IgM nephropathy; can we still ignore it. J Nephropathol.

[7] Myllymaki J, Saha H, Mustonen J, Helin H, Pasternack A (2003). IgM nephropathy: clinical picture and long-term prognosis. Am J Kidney Dis.

[8] Mubarak M, Kazi JI (2012). IgM nephropathy revisited. Nephrourol Mon.

[9] Connor TM, Aiello V, Griffith M, Cairns T, Roufosse CA, Cook HT (2017). The natural history of immunoglobulin M nephropathy in adults. Nephrol Dial Transplant.

[10] Kazi J, Mubarak M (2014). IgM nephropathy presenting as full blown crescentic glomerulonephritis: first report in the literature. Nefrologia.

[11] Swartz SJ, Eldin KW, Hicks MJ, Feig DI (2009). Minimal change disease with IgM+ immunofluorescence: a subtype of nephrotic syndrome. Pediatr Nephrol.

[12] Disciullo SO, Abuelo JG, Moalli K, Pezzullo JC (1988). Circulating heavy IgM in IgM nephropathy. Clin Exp Immunol.

[13] Border WA, Cohen AH (1983). Role of immunoglobulin class in mediation of experimental mesangial glomerulonephritis. Clin Immunol Immunopathol.

[14] Wyatt RJ, Julian BA (2013). IgA nephropathy. New Engl J Med.

[15] Zeis PM, Kavazarakis E, Nakopoulou L, Moustaki M, Messaritaki A, Zeis MP (2001). Glomerulopathy with mesangial IgM deposits: long-term follow up of 64 children. Pediatr Int.

[16] Westphal S, Hansson S, Mjornstedt L, Molne J, Swerkersson S, Friman S (2006). Early recurrence of nephrotic syndrome (immunoglobulin m nephropathy) after renal transplantation successfully treated with combinations of plasma exchanges, immunoglobulin, and rituximab. Transplant Proc.

[17] Betjes MG, Roodnat JI (2009). Resolution of IgM nephropathy after rituximab treatment. Am J Kidney Dis.

[18] Kanemoto K, Ito H, Anzai M, Matsumura C, Kurayama H (2013). Clinical significance of IgM and C1q deposition in the mesangium in pediatric idiopathic nephrotic syndrome. J Nephrol.

